# Could vectors’ fear of predators reduce the spread of plant diseases?

**DOI:** 10.1038/s41598-018-27103-y

**Published:** 2018-06-07

**Authors:** G. Tholt, A. Kis, A. Medzihradszky, É. Szita, Z. Tóth, Z. Havelda, F. Samu

**Affiliations:** 1 Plant Protection Institute, Centre for Agricultural Research, Hungarian Academy of Sciences, Herman Ottó út 15, Budapest, H-1022 Hungary; 2 Department of Systematic Zoology and Ecology, Faculty of Science, Institute of Biology, Eötvös Loránd University, 1/C Pázmány Péter Sétány, Budapest, H-1117 Hungary; 3 National Agricultural Research and Innovation Centre, Agricultural Biotechnology Institute, Szent-Györgyi A. út 4, Gödöllő, H-2100 Hungary

## Abstract

Predators influence the behaviour of prey and by doing so they potentially reduce pathogen transmission by a vector. Arthropod predators have been shown to reduce the consumption of plant biomass by pest herbivores, but their cascading non-consumptive effect on vector insects’ feeding behaviour and subsequent pathogen transmission has not been investigated experimentally before. Here we experimentally examined predator-mediated pathogen transmission mechanisms using the plant pathogen Wheat Dwarf Virus that is transmitted by the leafhopper, *Psammotettix alienus*. We applied *in situ* hybridization to localize which leaf tissues were infected with transmitted virus DNA in barley host plants, proving that virus occurrence is restricted to phloem tissues. In the presence of the spider predator, *Tibellus oblongus*, we recorded the within leaf feeding behaviour of the herbivore using electrical penetration graph. The leafhopper altered its feeding behaviour in response to predation risk. Phloem ingestion, the feeding phase when virus acquisition occurs, was delayed and was less frequent. The phase when pathogen inoculation takes place, via the secretion of virus infected vector saliva, was shorter when predator was present. Our study thus provides experimental evidence that predators can potentially limit the spread of plant pathogens solely through influencing the feeding behaviour of vector organisms.

## Introduction

Predation can induce important density and trait mediated effects on prey populations which have the potential to cascade through entire food webs and lead to ecosystem-wide effects^1,2^. In addition to predation (where the prey organism is consumed) it has been demonstrated that predators are able to influence various physiological, morphological and behavioural traits of their prey^3^ through various non-consumptive effects (NCEs). Behavioural traits such as vigilance, refuge seeking and fleeing are well studied predator avoidance mechanisms^4^ and are often in a trade-off relationship with fitness correlates: consumption, growth and mating success^5^. In herbivorous insects the indirect trait mediated effects of predators has been repeatedly shown to reduce herbivory and consequent plant damage; examples of this include the effect of spiders on the feeding of grasshoppers^6^, beetles^3^ and caterpillars^7^.

**Figure 1:**
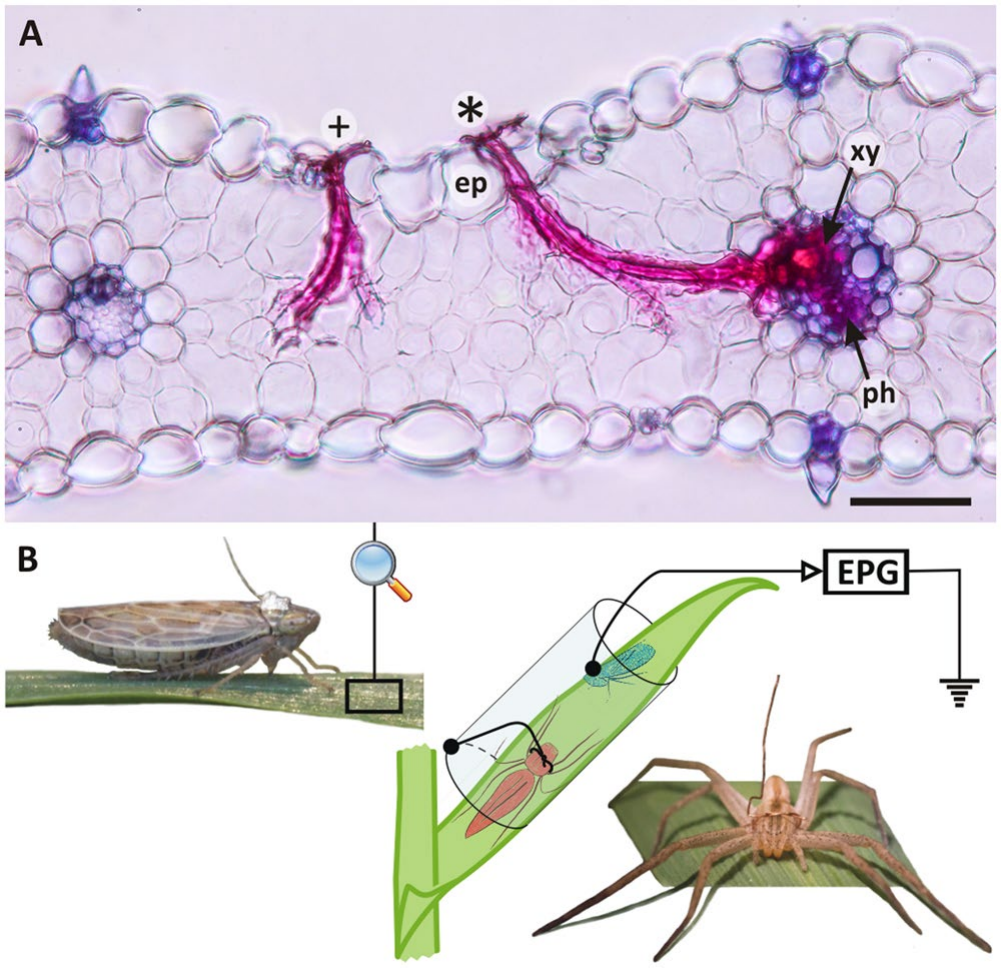
(**A**) Salivary sheaths produced by *Psammotettix alienus* in barley leaf. (**B**) Experimental setup to study the ‘Plant – leafhopper – spider’ model system. (**A**) Light microscopy image of salivary sheaths (SS) produced by *Psammotettix alienus* in barley leaf. Branches of the SS marked with a (+) at its start in the epidermis (ep) terminated in mesophyll tissue, while some of the branches of the SS marked with (*) terminated in transport tissues, phloem (ph) and xylem (xy). Scale bar = 200 μm. (**B**) Stages of penetration behaviour of the leafhopper *P*. *alienus* were observed on barley plants under predation risk by a confined spider (*Tibellus oblongus*) using an EPG device.

Sap feeding invertebrates are specialist herbivores which feed deep into the plant’s tissues. Although it is likely that the sap feeder guild of insects have undergone similar evolutionary pressures to avoid predation, studies of NCEs on sap feeders have been limited to movement and escape behaviours and were missing information about the actual feeding process. Sap feeders largely belong to the Homoptera and within this group, salivary sheath (SS) feeders produce a gel-like saliva, which lubricates and facilitates stylet movement in order to reach the plant’s target tissues^8,9^. The unit of the whole feeding process is a ‘penetration’, during which the stylets stay in the plant tissues^8^. After feeding the SS remains as an artefact within the leaves (Fig. 1A). SS feeders usually consume the sap from the plant’s transport tissues, the xylem and phloem^10^. During phloem ingestion nutrients are extracted from the phloem fluid, which is very rich in carbohydrates but deficient in others. This is a feeding environment of high osmotic concentration, which requires frequent drinking and makes homopterans sensitive to desiccation^11^.

In SS feeders predator vigilance and escape potential might be compromised during phloem location. This is because it is often a long process, with many trial and error elements and re-starts^12^, thus penetrations often end prematurely without reaching the phloem (Fig. 1A). Accessing deeper plant tissues requires longer time and more energy. The withdrawal of the stylets also takes longer from deeper positions, therefore SS feeders will not be able to respond quickly to predation risk in the more advanced penetration phases. Evolutionarily, SS feeders have to balance predator avoidance against their minimal nutritional requirements when engaged in feeding behaviour. In general, this suggests that longer feeding times increase exposure to predators, while delaying or shortening feeding times raises the risks of desiccation and starvation^11^.

Homopteran insects are the most important vectors of various plant diseases. For instance, 90% of known plant viruses are transmitted by this group^10,13^. In the case of all circulative viruses, but even in some of the stylet borne viruses, there is a strong coevolved relationship between the pathogen and its vector, and transmission would be impossible without the vector’s specific mode of feeding. In leafhoppers, different phases of the penetration are closely related to the pathogen transmission process. Infections are often limited to certain tissue types and the mode of feeding on these tissues affects transmission. For example, in the case of a phloem-bound pathogen, only the secretion of watery saliva of an infectious vector into the phloem can lead to pathogen inoculation, and vectors can only acquire the pathogen via ingesting phloem sap from an infected host plant^10,13^. If the complex sap feeding behaviour is responsible for the acquisition and inoculation of plant pathogens, then the NCEs of predators has a role of particular importance, as indirect trait mediated effects may not only impact the level of herbivory, but may also interrupt specific steps in the feeding process and thereby suppress the transmission and epidemic of plant diseases^14^.

We employed a ‘plant – leafhopper – spider’ model system (Fig. 1B) to quantify changes in sap feeding behaviour when a predator was present. To achieve a fine resolution, and detect different phases of the feeding process, electrophysiological observations of penetration behaviour were conducted in microcosms with the leafhopper *Psammotettix alienus* (Dahlbom) (Cicadellidae) on barley plants. *P*. *alienus* is an important pest of various cereal crops in Europe and Asia and is the only known vector of Wheat Dwarf Virus (WDV), which can cause severe losses in cereal crops^15–17^. WDV is a persistent circulative virus, which after ingestion via infected plant sap, concentrates in the salivary gland of *P*. *alienus* and is then able to re-inoculate other plants via excreted saliva. It has been assumed that circulative viruses are mostly phloem restricted, therefore the vector needs to reach the phloem in order to transmit the infection^17,18^, but no direct evidence has been published so far for the case of WDV. We have previously studied the NCEs of the ambush spider predator *Tibellus oblongus* (Walckenaer) (Araneae: Philodromidae) on the movement behaviour and the feeding frequency of *P*. *alienus* in a coarse resolution mesocosm study, where predator-prey encounters (including the possibility of predation) were allowed^19^. In the present microcosm study we used confined predators which were unable to reach and consume their prey (Fig. 1B), in order to directly measure non-consumptive effects on finer details of the feeding behaviour.

Electrical penetration graph (EPG)^20^ measurements are the most appropriate method to obtain detailed live data about the complex penetration process of sap feeders. In an EPG measurement insects and plants are part of an electrical circuit where fluctuating voltage changes represent the different phases of the penetration process^20,21^. Analysis of an EPG curve produces a time sequence which specifies the different phases and their duration as well as the type of tissue the leafhopper is feeding on. To adapt EPG techniques, for the first time, to monitor changes in the penetration process resulting from predator NCEs, we applied a robust and functional categorization of the recorded EPG waveforms^15^ (see Materials and Methods for correspondence between the technical and present functional categorization). Four categories were identified: (1) “travel phase”, which includes the piercing of the epidermis, SS production and the stylets’ travel through non-target tissues. In this phase, accessory feeding is possible from mesophyllum cells which have been reported to be brief events^22^. (2) “drinking phase” which involves the active ingestion of the xylem sap, which is essentially water. (3) “phloem preparation phase” this occurs when the phloem is reached and involves the secretion of a defined volume of watery saliva into the phloem; carried by the saliva, virus inoculation takes place in this phase^22,23^. (4) “phloem ingestion phase” which is always preceded by the preparation phase, involves the ingestion of phloem sap assisted by phloem pressure. Virus acquisition occurs in this phase. This stage is largely a passive process which can take a considerably long time, up to several hours.

Previous studies have documented the movement responses of sap feeders to the presence of predators and parasitoids. These studies concluded that activity changes due to NCEs can either increase or decrease pathogen spread^24,25^. Comparative experiments^26^ show that the mode of transmission and the type of sap feeding have a decisive role in the outcome. Predators that induced frequent vector movement caused a suppressed spread in the case of a circulative persistent virus (Cucurbit aphid-borne yellows virus, CABYV), because this transmission type requires a period of longer feeding. Whereas, in the same study, predators increased the spread of a non-persistent stylet-born virus (Cucumber mosaic virus, CMV) because it is more easily transmitted during short feeding sessions^26^. Modelling studies^14,27^ have highlighted that in addition to the density mediated effects of predators, vector movement, migration and the modification of the feeding process are important parameters when assessing the cascading effect of predator NCEs on pathogen spread. Of these factors, the NCEs of predators on sap feeding behaviour have not been investigated experimentally.

Our study focused on whether balancing predator avoidance and feeding constraints led to any significant change in the feeding process of a sap feeding insect. Our objective was to show whether modified sap feeding had the potential to affect pathogen transmission in vector insects. We studied how different penetration phases, linked to pathogen transmission in different ways, changed with predation risk. We argued that if such changes were demonstrated, then predictions on how NCEs might affect the vectoring capability of these insects could be made. We assessed the location of virus DNA in the plant tissues following virus infection, which allowed us to identify the key penetration phases involved with pathogen inoculation and acquisition. We showed that virus DNA is only found in the phloem following infection. A novel application of the EPG technique in an interspecific scenario made it possible to follow the penetration process in real time while subjects in the predator treatment were exposed to predation risk and in the control treatment could feed without predator presence. We demonstrated a significant and differential predator induced change in penetration behaviour which resulted in a feeding pattern that decreases the likelihood of virus transmission in this agriculturally important tri-trophic model system.

## Results

### Feeding activity

**Figure 2:**
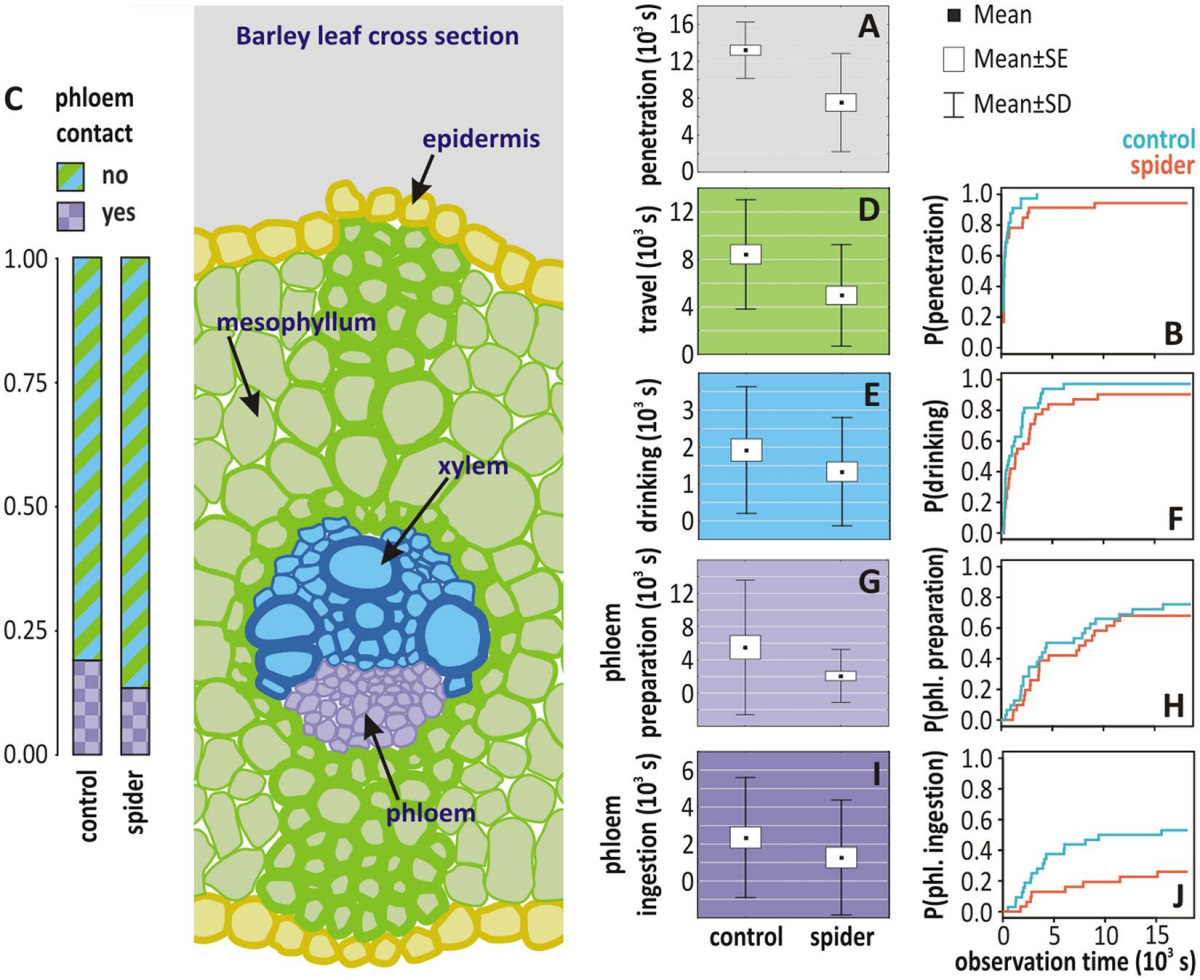
The effects of predator presence on penetration. The effect of predator (spider) presence on penetration phase durations and their probability to start, referenced against (colour coded) a leaf cross section where the phases take place. Between treatment comparison of total penetration duration and the probability of occurrence, respectively, for the following phases: travel (**D**,**B**), drinking (**E**,**F**), phloem preparation (**G**,**H**), phloem ingestion (**I**,**J**) and for complete penetrations (**A**,**B**). Boxplots depict mean, S.E. and S.D. derived from the raw data. Histogram under (**C**) gives the proportion of penetration events that either reached or did not reach phloem in the treatments.

In order to detect the leafhopper’s response to a spider’s presence while feeding, we first analysed changes in the number and total duration of penetration events (n = 716). The number of penetrations was only marginally lower in the predator treatment than in the control group (negative binomial GLM: z = −1.923, P = 0.054), but, considering the total duration of the behaviour, leafhoppers spent significantly less time engaged in penetration activities in the predator treatment than in the control (Table 1, Fig. 2A). Overall, 16.2% of all penetrations reached phloem tissues (successful recordings of a phloem preparation phase during the event); while in 83.8% of the penetrations the stylets were withdrawn before phloem contact (Fig. 1A). In the predator treatment fewer penetrations reached the phloem tissue (12.8%) in comparison to the control treatment (18.6%) (χ^2^ = 4.29, d.f. = 1, P = 0.038; Fig. 2C).

**Table 1:** Summary of statistical results.

	Total duration of event*				Delay until first occurrence of event**		
	model	test statistics	d.f.	P	HR	z	P
Penetration event	GLS	F = 21.62	1,61	0.001			
Travel phase	GLS	F = 11.35	1,60	0.001	0.7	−1.985	0.047
**Drinking phase**							
occurrence^†^	BGLM^§^	z = −1.02		0.309	0.66	−2.173	0.029
duration^‡^	gGLM^¶^	t = −1.23	58	0.224			
**Phloem preparation phase**							
occurrence^†^	BGLM^§^	z = −0.64		0.519	0.81	−0.836	0.4
duration^‡^	gGLM^¶^	t = −2.51	44	0.016			
**Phloem ingestion phase**							
occurrence^†^	BGLM^§^	z = −2.18		0.029	0.38	−2.082	0.037
duration^‡^	gGLM^¶^	t = 0.26	24	0.794			

Models and their results for the effect of predator presence on penetration phase durations and their probability to start. *Duration (s) EPG parameters were tested by linear models. **Probability of occurrence of events was tested by Cox proportional hazard model. ^†^Tested on all cases. ^‡^Tested only on non-zero cases. ^§^Bernoulli GLM. ^¶^gamma GLM.

### Penetration phases

To detect possible NCEs on the different penetration phases we considered two main parameters, the total duration of a penetration phase and the delay until first occurrence of a phase. All statistical test results pertaining to these parameters are presented in Table 1. Starting time (delay) and total duration of the phases were calculated from the raw waveform data from the EPG measurements. Descriptive statistics for the waveform data are given in Supplementary Table 1. Overall, leafhoppers spent significantly less time in the travel phase of the penetration process, when predators were present (Fig. 2D). The spider’s presence also resulted in an increased delay until the occurrence of the first travel phase event (which is equal to the delay until first penetration) i.e. in the predator treatment there was a lower probability of starting a penetration than in the control (Fig. 2B).

Unlike the travel phase, the overall time engaged with the drinking phase did not differ between the predator and the control treatments (Fig. 2E). The drinking phase and subsequent deeper phases were not observed in a considerable number of trials, resulting in an inflation of zero-duration cases for the given phase. For these phases we applied zero-added models^28^, which tested; a) the duration of an event for the cases where the event occurred and b) for all cases the frequency of occurrence (ratio of 0–1 values). In the case of drinking, predator presence did not influence either aspect of this phase but the time delay for leafhoppers to start their first drinking phase was longer in the predator treatment (Fig. 2F).

We tested predator effect on the duration of the phloem preparation phase (Fig. 2G). When this phase occurred it was significantly shorter in the predator treatment. However, neither occurrence probability nor the time until the first occurrence of the phloem preparation phase (Fig. 2H) differed between the treatments.

Phloem ingestion was significantly influenced by predator presence (Fig. 2I), yet the zero-added model suggests a response that differs from that in the preparation phase. The probability of the occurrence of phloem ingestion significantly depended on predator presence. In the predator treatment eight of 31 leafhoppers initiated phloem ingestion, while in the control group this phase occurred in 17 out of 32 leafhoppers. The time delay to initiate phloem ingestion was significantly longer in the predator treatment (Fig. 2J), but the total duration of this phase did not differ significantly between the treatments.

Saliva secretion into the phloem (phloem preparation phase) is essential and always precedes phloem ingestion. We predicted that predator presence may interrupt this process, leading to the preparation phase occurring while the ingestion phase is absent. We tested for this effect by comparing the ratio of the frequency of preparation phase events to ingestion phase events in a quasibinomial GLM. A smaller proportion of preparation phase events were followed by ingestion event under predation threat in comparison to the control (z = 2.122, P = 0.040).

**Figure 3:**
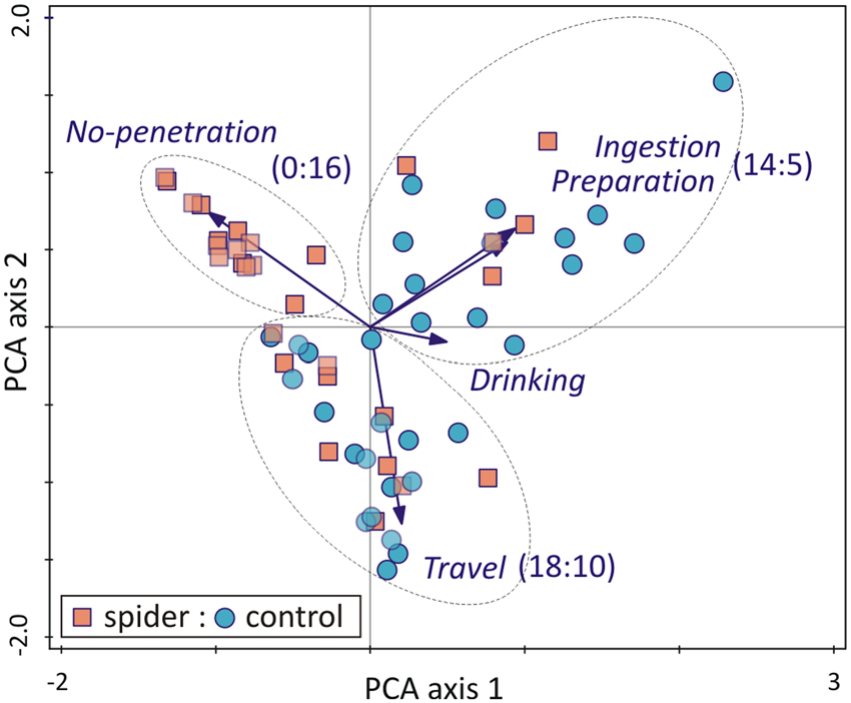
Principal component analysis (PCA). Principal component analysis (PCA) biplot of EPG observations (symbols) on the variables (arrows) of total duration of penetration phases including the “phase” no-penetration. The first two axes accounted for 70% of variance. Observations that can be associated with a variable or variable group were delineated (dotted ellipses) and the ratios of observations falling in spider vs. control treatment in such groups are given.

The multivariate pattern of penetration phase durations among leafhoppers in the predator and control treatments was analysed by PCA ordination (Supplementary Table 2). The ordination plot (Fig. 3) revealed three groups of individuals (we tested for the validity of this grouping by MRPP, T = −30.61, P < 0.0001). Individuals that engaged only minimally with feeding activities belonged to the ‘no-penetration group’, and were without exception from the predator treatment. In contrast, in the ‘ingestion group’ control individuals were in a c. 3:1 majority to predator treatment individuals. The third group was engaged with travel phase activities, with individuals in the control treatment in clear majority (Fig. 3).

### WDV localization in leaf tissues

**Figure 4:**
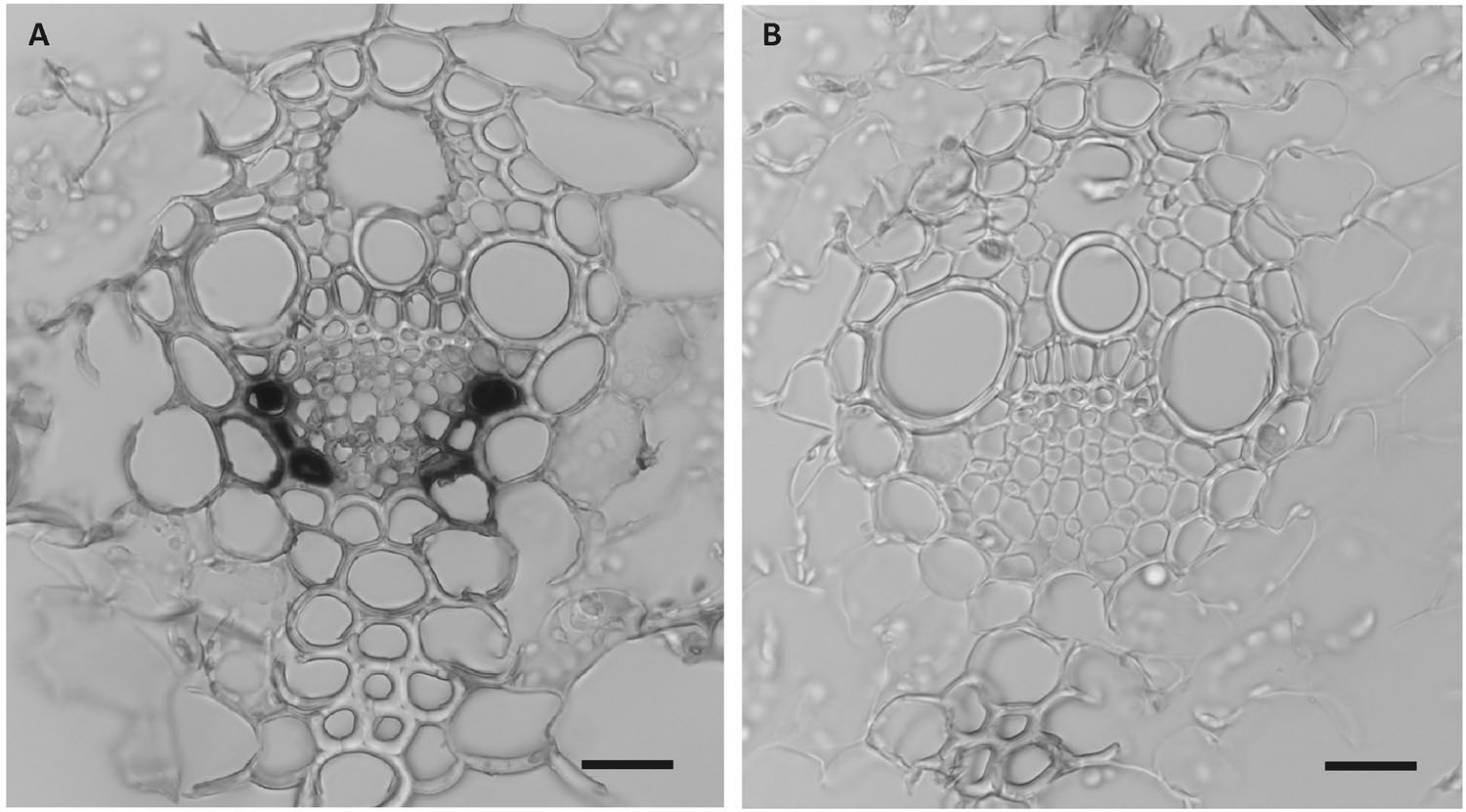
Barley leaf cross sections. WDV mRNA hybridization on virus infected **(A)** and control **(B)** leaves. (Scale bars = 20 μm.)

To assess how altered feeding activity might affect virus transmission, the previously unknown localization of WDV had to be confirmed. We examined the virus DNA localization in WDV infected leaf tissues, as well as mock treated controls, by *in situ* hybridization (Fig. 4). This revealed that four weeks after virus infection in the treated samples (n = 12) WDV was clearly detectable only in the phloem tissues. No signals were detected in the control samples (n = 10).

## Discussion

### Changes in feeding activity

In the presence of a spider leafhoppers decreased the duration of penetration activities and delayed their first penetration event. As a consequence of this, the feeding habits of the leafhoppers were altered, and overall, spent less time feeding. A decrease in feeding activity is perhaps the most common response to predation risk, since antipredator behaviours, such as vigilance^29^, refuge seeking^30^, defensive drops and jumps^11,19^ are in a trade-off relationship with feeding. Shorter feeding times lead to a reduced uptake of plant sap, because in sap feeders the ingestion of plant fluids occurs at a relatively constant rate^31^. For optimal nutrition the ingestion of high sap volume is necessary due to the need to extract limiting nutrients from their diet of mostly pure plant sugar solution^32,33^. As a side-effect, phloem sap feeding also poses the threat of osmotic imbalance and requires frequent drinking as a countermeasure^34^. EPG studies have revealed that within a penetration event drinking phases often occur repeatedly and intermittently with phloem ingestion phases^15,22^ making the penetration process overall longer. Furthermore during phloem ingestion, animals must contend with the high turgor pressure of the plant sap which can expel the stylets^35^. In SS feeding leafhoppers, this is prevented by opening the serrated end of the stylets fascicula when phloem tissues are reached^36^. Feeding on phloem sap therefore leads to not only prolonged exposure to predators, but also delayed escape possibility. In our previous mesocosm experiment^19^ and in the present electrophysiological observations we show that predator presence has altered the balance between the non-feeding and feeding periods.

### Distribution of penetration phases

From this and previous studies it is clear that feeding periods are shortened as a response to predation risk. One of the most important advantages using EPG techniques is that it gives us a uniquely detailed picture of crucial, but otherwise hidden behavioural responses, which help us to understand the underlying optimization processes in the feeding behaviour of sap feeders under NCEs. Although EPG is widely used in insect-plant interaction and in vector studies, we have shown for the first time that it can be a powerful tool in behavioural ecological studies of interspecific interactions.

If less time is spent by the herbivore penetrating plant tissues as a consequence of NCEs, sap feeders are expected to optimize the distribution of different penetration phases taking into account factors such as physiological limitations and risk from predators. Due to high desiccation risk, drinking is the most obligatory phase^31^ and, supporting our original predictions, neither the probability of its occurrence nor the overall duration of drinking events was affected by predation risk, even though the first occurrence was delayed when a spider was present.

The travel phase and phloem preparation phase were both of reduced duration as a result of predator NCEs. Based on the assumption that withdrawal of the stylets is easier during these phases we predicted that these behaviours would be less affected by predation risk in comparison to phloem ingestion, when withdrawal of the stylets is more difficult. Despite this, these predictions were incorrect. There was a roughly similar trend in reduced duration in the travel phase and deeper penetration events. During the travel phase SS feeders try to locate and puncture leaf veins while continuously secreting gelling saliva. Producing SS has a time and energy cost, and having made such an investment, this may lead to leafhoppers becoming progressively more risk-prone^29^. In aphids, the value of SS has been demonstrated as they would compete for SS made by other individuals, which then was used to locate the phloem tissues quicker^37^. In comparison to aphids, which only briefly sample epidermis and mesophyll cells, phloem feeding leafhoppers cause more damage and can ingest a significant amount of mesophyll cell content which is likely to be an important part of their diet^15,22,38^. As such, mesophyllum feeding during the travel phase might provide an alternative, albeit a different quality food resource. The possibility to choose between resources that are different both nutritionally and in riskiness might broaden the repertoire of antipredator strategies and may play a role in how the distribution of penetration phases is optimized in our model system.

The deepest penetration phase, phloem ingestion, had fewer occurrences in the predator treatment. The preparation phase became shorter as a result of a spider’s presence and was less often followed by ingestion. Surprisingly, when it did occur, the duration of phloem ingestion was not affected by NCEs. One could speculate that this penetration phase is a more conservative behaviour, but the relatively large variation in its duration (see Supplementary Table 1) contradicts this idea. Perhaps the phenomenon is connected to the diminished escape capability of leafhoppers during this phase, which is why the animals engage in this activity less often, but once it has started it might be more optimal to ‘keep a low profile’ on the approach of a visual predator than to engage in a handicapped escape activity.

Changes in feeding behaviour and escape activities imply that the potential predator is detected by the leafhopper. As we have seen, these behavioural changes are contingent on the phase of the feeding process. Simple behavioural observations, like the ones conducted in a mesocosm^19^, cannot clarify the details of what modalities are used for predator detection^8^, because only an overall behavioural response can be detected. The present EPG study was also designed for multimodal perception, in a way that all possible modalities of predator stimuli could reach the target animals and their separation was not possible. Leafhopper sensory biology in general can be regarded as an understudied area. The role of olfaction and visual stimuli has been reviewed by Backus^8^, but virtually all studies, similarly to the reviewed ones, scrutinized their role only in the context of host plant detection^39^. As an exception, the function of vibratory signals and stimuli has been recently shown in the mate finding of leafhoppers^15,40^. On the whole, the role of different sensory modalities in predator avoidance has not been explored yet in leafhoppers.

### Can altered feeding activity influence virus transmission?

Transmission (acquisition and inoculation) of plant pathogens usually occurs during vector feeding^41^, the speed of which largely depends on the pathogen’s behaviour within the vector. In the case of non-circulative stylet-born transmission, virus contaminated mouthparts can inoculate plants quickly, within the first few minutes of the penetration. Conversely, SS feeder leafhopper borne viruses, including WDV, are mostly transmitted in a persistent circulative manner^42^.This means that the virus is able to concentrate in the salivary glands of the host and the inoculation occurs when the saliva is secreted into the transport tissues and acquisition occurs when the sap is ingested^43^. In a non-circulative case the transmission efficiency of CMV by *Aphis gossypii* Glover was increased if aphids made more frequent and shorter penetrations^44^. For circulative pathogens transmission success is dependent both on the frequency and duration of certain penetration phases and usually requires a more specific and an “above threshold duration” contact between vector and host plant. In a circulative case an increased duration of phloem contact was required to significantly enhance the inoculation efficiency of the Tomato yellow leaf curl virus by *Bemisia tabaci* (Gennadius)^45^. Similarly a longer vector inoculation time resulted in increased Maize chlorotic dwarf virus transmission rates^42^. To quote a non-virus example, if penetration was interrupted after the phase equivalent to the travel phase, then a phloem restricted *Spiroplasma* could not be inoculated, because for successful inoculation phloem-bound phases were required^46^.

In *P*. *alienus* we showed direct evidence that WDV is a phloem-restricted virus in the *in situ* hybridization experiments. Present studies lend strong support to the hypothesis that perceived predation risk decreases the probability of virus transmission in our model system. The EPG analysis demonstrated that both the phloem ingestion phase (responsible for virus acquisition) and the phloem preparation phase (responsible for virus inoculation) are negatively affected by predator NCEs. In addition, phloem ingestion occurred with an increased latency and was also less frequently observed. When a predator is present it can be predicted that, resulting from predator NCEs, leafhoppers will acquire the virus less frequently. In order to quantitatively assess how shortened phloem preparation phases influence virus inoculation we would need studies that specifically address what threshold duration is needed for the inoculation of the virus or whether other dynamics apply to this process.

### Potential agricultural implications

The effect of predators on the vectoring efficiency of their potential prey has important agricultural implications. Vectors need to recolonize annual crops every year and that, at least partly, resets the cycle of pathogen epidemics. For this reason reservoir areas play an important role in the dynamics of annual crop-pathogen systems^47^. In the case of *P*. *alienus* and WDV, grassy edges, fallows and self-sown areas play a key role in the recolonization of cereal fields^16,48^ and in the maintenance of a virus reservoir^49^. To estimate predator effect accurately, we have to consider the spatial and temporal dynamics of the vector population, habitat specific predator presence and both the consumptive and non-consumptive effects of predators.

Management (e.g. harvest, stubble harrowing) induces migration to less preferred habitats and causes drops in *P*. *alienus* density^50^. Incidentally, natural enemies, including *T*. *oblongus*, are in higher densities in these marginal areas than in crop fields^51^ and can conduct their predatory and non-consumptive effects at a vulnerable stage of the vector populations. Predators can induce increased movement of vectors which may result in a reduction of disease risk when extended feeding bouts are required for pathogen transmission^14,25,26^. The complex interactions between predator presence, the feeding process and plant (or leaf) relocations are hard to simulate in simplistic microcosm experiments. Our experiments presented the herbivores with constant predator cues. At high predator densities and in cases when predators, like spiders, leave olfactory cues, the assumption that the herbivores are exposed to constant or frequent predator cues in certain field situations is not unrealistic. Here we argue that if the right conditions met then some vectoring modes are more prone to natural enemy NCEs.

The persistent circulative transmission of WDV by *P*. *alienus* fits the manner of pathogen transmission that can be effectively disrupted by NCEs, because leafhoppers specifically have to reach deep regions of the plant tissues, the phloem in specific, to acquire or inoculate the virus. As a result of NCEs, increased movement rates and shortened feeding times of *P*. *alienus* have also been shown in a previous study^19^, whereas a reduction in the components of *P*. *alienus* feeding behaviour that lead to virus transmission has been demonstrated in the present experiments. Our study highlights that trait mediated effects of predators induce significant changes in sap feeding behaviour which in combination with other behavioural interactions and the density mediated effect of predators can potentially limit the spread of plant pathogens.

## Methods

### Experimental plants and animals

Throughout our investigation, leafhoppers were reared on and experimental trials were conducted on spring barley (*Hordeum vulgare*). Experimental leafhoppers were obtained from our stock population (originating from the field several generations ago), spiders were collected from the field, then kept under artificial conditions for several weeks. For full details see SI Materials and Methods.

### Microcosm setup and EPG recordings

The penetration process was monitored using the EPG technique within a microcosm enclosed in an open ended glass tube (80 mm length, 30 mm diam., fixed on a stand in a half-vertical, 45° position). The microcosm contained (i) a section of the barley leaf; (ii) the leafhopper attached to the EPG electrode wire; and in the case of predator treatment (iii) a spider, also leashed on a wire fixed at the other end to the tube, ensuring that it could not position itself within 30 mm of the leafhopper. The second leaf of the potted barley plant was used consistently, it was pulled through the tube and gently stretched along the inside wall allowing the animals access to the upper surface (Fig. 1B). Microcosm setups were made for each of every EPG channels and were placed inside the Faraday cage of the EPG device.

Leafhoppers were removed from the stock for EPG recordings from their rearing plant an hour before in order to standardize their starvation level. Removal of an animal occurred by random choice and animals were alternately assigned to the control or to the spider treatment. At the end of the starvation period we anesthetized leafhoppers with CO_2_ and attached a 30 mm long, 18 µm diameter gold wire, serving as the electrode, to the scutellum using silver glue. Spiders selected for the treatment were also anaesthetized in order to tie a loop knot of a thin copper wire (49 µm) around their cephalothorax between the second and third legs. When the spiders recovered they were introduced into the glass tube. The spider’s leash wire was fixed in a way that allowed natural movement and position, only restricting the movement range. The spider was always positioned in the lower end of the tube, while the leafhopper was in the upper half.

For the EPG recordings, we used a GIGA-4 DC and two GIGA-2 DC EPG devices (EPG Systems, The Netherlands) in parallel. Recording sessions were five hours long and eight channels were simultaneously recorded (4–4 treated and controls in random combinations). There were 10 sessions in total, n = 80 recordings, but – as a predetermined criterion – we excluded cases where either of the animals managed to release themselves from the wire. This left us with 31 treatment and 32 control cases. This sample size was determined to be similar to that of in a previous study^19^, where we have found significant effect of spider presence on the feeding behaviour of leafhoppers observed with traditional ethological methods in the same model system. For recordings and analysis we used Stylet + Data Acquisition and Analysis software (EPG Systems, The Netherlands). During waveform analysis the investigator was blinded to treatment group allocation by including this information in file metadata in a coded way.

### EPG parameter calculation

In our previous work we have described and classified the EPG waveforms of *P*. *alienus*^15^. In the current work we used the following simplified categorization of waveforms: “travel phase” covers all non-target tissue penetration phases (Ps1, Ps3 and PsMix waveforms); “drinking phase” corresponds to Ps2 waveform; “phloem preparation phase” corresponds to Ps4a waveform – which is an X-wave according to EPG studies on other homopterans and also biologically equivalent to E1 phase in aphids. Phloem preparation is an obligatory step which precedes phloem ingestion and in this phase watery saliva is released into the phloem vessels in order to suppress the plant’s defences^23^. The “phloem ingestion phase” corresponds to Ps4b waveform. We used the term “penetration” to indicate a period when the leafhoppers’ mouthparts were in the plant leaves (which also meant that the EPG circuit was closed and we had a non-zero signal). At the start of the trials and between any two penetrations we had “no-penetration phases” with a zero signal. This indicates an activity on the leaf surface other than penetration. For the statistical analysis we calculated the overall duration of the different penetration phases, the number of penetration events during the five hour observations as well as the latency (time delay), until the first occurrence of a given phase or penetration event. In the penetration phase we used the frequency of penetrations that occurred during a single observation as well as the frequency of preparation and ingestion phase events when attempting to examine whether their ratio is sensitive to predator presence.

### *In situ* hybridization of virus DNA in leaf tissues

We transferred WDV from infected stock plants to barley plants using *P*. *alienus*. Infection was subsequently confirmed by PCR. Samples for *in situ* hybridization were collected four weeks after inoculation and then tested by our previously described protocol^52^, details of which can be found in SI Materials and Methods.

### Statistical analysis

Our analyses were conducted in R v. 3.3.2^53^ and in PC-ORD v. 6.19^54^. We had three types of data: (a) time delay until the first occurrence of an event (penetration or given penetration phase), (b) event duration and (c) frequency of penetration events. In all cases we modelled these response variables as being dependent on two factors: predator treatment (control or spider) and leafhopper sex (male or female). Time delay data, in all cases, was modelled using the Cox proportional hazards (CPH) model and we treated cases where the given event did not occur as censored. The assumption of validity of proportional hazards were checked using the functions “coxph” and “cox.zph”, respectively, in the “survival” R package^55^. In the case of duration, we applied models from the linear family if normality could be achieved with simple transformation. In the case of penetration duration we applied square root transformation on the response variable and fitted generalized least squares (GLS) models (R package “nlme”^56^) with “varIdent” weight to control for unequal variances between the treatment groups. In the case of travel phase duration we fitted a linear model after square root transformation. For all penetration phase durations that were deeper than the travel phase, analysing data distribution revealed a zero-inflated distribution, attributable to the fact that in many penetrations the given phase did not occur at all. Therefore, for duration data of the drinking, phloem preparation and phloem ingestion phases zero-added gamma (ZAG) models were applied^28^ using the “glm” function of the “base” R package. Due to overdispersion, we fitted a negative binomial model on the number of penetrations with a log link. The fit of all linear and generalized linear models were checked by inspecting residuals and QQ plots. A multi-dimensional treatment of total duration of each penetration phase (including the phase “no penetration”) exhibited by the observed leafhoppers was achieved by Principal Components Analysis (PCA). We used a randomization test over 999 iterations to decide how many axes carry significant information by comparing the original Eigenvalues received for the axes against the Eigenvalues received after randomization of variable values (columns) in the data matrix^54^. We tested whether the observed clustering of observations comprise groups in a sense that the within group distances are smaller than could be expected by chance. This was tested by Multi-response Permutation Procedure (MRPP), which calculates whether the weighted mean within group distances are smaller than could be expected in permutated datasets, giving the test statistics ‘T’^54^.

### Ethics statement

All applicable international, national, and/or institutional guidelines for the care and use of animals were followed. All procedures performed in studies involving animals were in accordance with the ethical standards or practice of the institution at which the studies were conducted.

### Data availability

The complete dataset of the experiment is available at the following link: https://figshare.com/s/07bd26f7310eb06a5aa2.

## Electronic supplementary material

Supplementary Information
