# Supplementary material for: Could vectors’ fear of predators reduce the spread of plant diseases?
Source: Sci Rep. 2018 Jun 7;8:8705. doi: 10.1038/s41598-018-27103-y (PMC5992157; doi:10.1038/s41598-018-27103-y)
Supplement: Supplementary file 1 — Supplementary Information [file 41598_2018_27103_MOESM1_ESM.pdf]

Could vectors' fear of predators reduce the spread of plant diseases?

Author information

Tholt, G.[a,b], Kis, A.[c], Medzihradszky, A.[c], Szita, É.[a], Tóth, Z.[a], Havelda, Z.[c] and Samu, F.[a,*]

[a] Plant Protection Institute, Centre for Agricultural Research, , Hungarian Academy of Sciences, Herman Ottó út 15, Budapest, H-1022, Hungary.
[b] Department of Systematic Zoology and Ecology, Faculty of Science, Institute of Biology, Eötvös Loránd University, 1/C Pázmány Péter Sétány, Budapest, H-1117, Hungary.
[c] National Agricultural Research and Innovation Centre, Agricultural Biotechnology Institute, Szent-Györgyi A. út 4, Gödöllő, H-2100, Hungary

Supplementary Information

Methods

Experimental plants and animals. Spring barley (*Hordeum vulgare*, cv. Mv. Conchita) plants were used in all experiments and to produce breeding stocks of leafhoppers. Seeds were individually planted in peat pellet (Jiffy-7; Jiffy Products International B.V., Netherlands), then, after successful germination were transferred into 0.2 l pots filled with greenhouse soil mixture ('Jóföld' type 'Balkonláda'; Pax96 Ltd., Kecskemét, Hungary). In all experiments, plants were used at the three leaves stage. Plants for *P. alienus* stock populations were grown in the same soil mixture until the advanced tillering stage; the plants were then transferred to 1l pots and kept under glasshouse conditions.

Our *P. alienus* stock population (consisting of approx. 1500 individuals continuously) have been kept for several years in a climate chamber (L/D: 16/8 h, 23/ 16°C regime) and leafhoppers develop on potted barley constrained by a fine nylon mesh. The original leafhopper stock was collected from self-sown barley fields near Páty, Hungary (47°30'23.25"N; 18°48'26.30"E) in 2013. These stock populations are refreshed every year by collecting leafhoppers from the same area. This population of leafhoppers is kept in quarantine for a generation before mixing with the stock population. Prior to experiments a cohort of leafhoppers, in the last larval instar, are selected and kept separately until adulthood. Sample specimens from the stock population were checked by PCR to be free from WDV infection. The leafhoppers used in experiments are collected randomly from this selected stock to ensure that experimental animals are approximately of the same age.

T. oblongus juveniles were collected from fallow and grassland areas near Budapest, Hungary (47°32'58.94"N, 18°55'48.81"E) using a sweep net, and were kept in the same conditions as the leafhoppers. Collected animals were housed in individual plastic vials with a moistened plaster of Paris base, and fed *ad libitum* with *Drosophila melanogaster* (Meigen) twice a week. Prior to experimentation, spiders were kept in the laboratory for at least four weeks, during which they received the *Drosophila* diet and were completely separate from any leafhopper or other prey. During this period spiders usually moulted at least once. Species identity of juvenile *T. oblongus* can

be determined with high certainty; nevertheless, random individuals were selected and reared to adulthood. Species identity was checked and confirmed by the microscopic examination of their genitalia.

Light microscopy images. For demonstrative purposes we have produced cross section images of barley leaves containing leafhopper salivary sheaths. The plants used for these observations were grown in the same conditions as plants used for the EPG experiments. Three to four leafhoppers were consecutively placed on plant leaves in microisolators (5 cm long, 3 cm in diameter, plastic tube with nylon mesh wall). After 24 hours the leafhoppers were removed from the plants, the plant leaves were cut off and placed into an Eppendorf tube filled with McBride solution. The leaves were left for two hours at room temperature. After staining, samples were washed three times using PBS, and then transferred into tubes filled with clearing agent which contained 87.5% glycerine and pure lactic acid (1:1 vol/vol). The tubes were placed into a dry block thermostat at 60°C for approximately three hours or until the sample had completely clarified. Samples were then washed three times in PBS again and were cut by hand using a sharp razor blade. Sections with stained salivary sheaths were placed on microscopic slides in 87.5% glycerine then covered with a coverslip. The samples were examined and photographed immediately.

In situ hybridization of virus DNA in leaf tissues. To produce WDV-infected plants, we used the same method as in our previous work [55]. Virus-free adult *P. alienus* individuals were placed for seven days on WDV-infected barley plants. Infected leafhoppers were then moved onto 2-3 week old test plants using two micro-isolator chambers; each chamber contained three insects, for a week. The insects from each chamber were pooled and PCR tested for the presence of WDV using the Phire Animal Tissue Direct PCR Kit (Thermo Fisher Scientific, Vilnius, Lithuania) and WDVrepDetF (CGCCTTGGACTCTCTTCGCAC) and WDVrepDetR (GACGGATAGACCATTCAAACG) primers. Plant infection was tested after four weeks by PCR using the same method.

In situ hybridizations were performed as described by Medzihradszky, et al. [50]. Leaf samples were collected four weeks after inoculation. Overall 12 WDV infected and 10 control samples were tested from 2-2 plants in two biological replicates. The full length WDV genome, in complementary-sense orientation, was cloned into a pBlueScript II KS (+) vector from the agroinfectious WDV clone pPZP201WDV1,5MER, as described in Kis, et al. [55], with HindIII digestion. The DIG labelled RNA probe (detecting Rep/RepA) was synthesized with T3 polymerase from a PCR amplified virus sequence, using the T3 and T7 primers.

Supplementary Table 1. Recorded waveform types during the EPG observations and their statistics in the two treatments (spider, control). Event is a continuous period for which the waveform was recorded (in seconds), total duration is the total duration of events during an EPG recording session, and frequency is the number of occurrences.

Waveform*	Treatment	Mean(event frequency)	SD(event frequency)	Mean(event duration)	SD(event duration)	Mean(total duration)	SD(total duration)
np	control	12.91	8.935	371.00	832.105	4788.21	3049.750
np	spider	9.77	7.056	1048.88	2655.629	10252.00	5317.987
Ps1	control	25.03	14.363	77.21	134.631	1932.59	1276.614
Ps1	spider	17.94	11.744	73.33	71.822	1315.21	813.011
Ps2	control	7.81	5.509	245.59	501.699	1918.64	1713.719
Ps2	spider	5.58	4.233	238.98	371.927	1333.67	1464.929
Ps3	control	6.81	5.083	502.31	661.062	3422.01	2939.317
Ps3	spider	4.90	4.475	514.31	785.830	2521.77	3132.555
Ps4a	control	2.47	2.328	222.82	243.809	550.09	809.970
Ps4a	spider	1.26	1.264	164.34	161.850	206.75	318.444
Ps4b	control	1.41	1.643	1652.57	1850.831	2323.93	3249.884
Ps4b	spider	0.39	0.715	3206.52	3375.536	1241.23	3110.394
PsMix	control	4.53	3.473	676.31	1358.779	3064.54	4069.874
PsMix	spider	2.68	3.059	421.82	414.825	1129.38	1723.318

*Waveform nomenclature after [15].

Supplementary Table 2. Results of PCA analysis of EPG observations on total duration variables of the respective event types: no-penetration, travel phase, drinking phase, phloem preparation phase and phloem ingestion phase.

Axis	Eigenvalue	% of Variance	Cum.% of Variance	Broken stick Eigenvalue	Eigenvalues from randomizations Minimum	Average	Maximum	P
1	1.858	37.168	37.168	2.283	1.1406	1.361	1.7585	0.001
2	1.644	32.887	70.056	1.283	0.97367	1.1471	1.3752	0.001
3	0.968	19.366	89.422	0.783	0.7874	0.98982	1.155	0.662
4	0.529	10.578	100	0.45	0.6103	0.83644	1.0089	1
5	0	0	100	0.2	0.30575	0.66568	0.87557	1
